# Diagnostic accuracy of high-frequency ultrasound for cutaneous neoplasms: a narrative review of the literature

**DOI:** 10.1007/s00403-024-03179-7

**Published:** 2024-06-21

**Authors:** Catherine Grace P. Hobayan, Ashley N. Gray, Margo F. Waters, Layna A. Mager, Sonja Kobayashi, Ellen W. Essien, Catherine A. Ulman, Benjamin H. Kaffenberger

**Affiliations:** 1 The Ohio State University College of Medicine, Columbus, OH USA; 2 Department of Dermatology, The Ohio State University, 540 Officecenter Place, Suite 240, Columbus, OH 43230 USA

**Keywords:** Cutaneous neoplasm, General dermatology, Medical dermatology, Oncology, Pathology, Ultrasound

## Abstract

High-frequency ultrasound has been used to visualize depth and vascularization of cutaneous neoplasms, but little has been synthesized as a review for a robust level of evidence about the diagnostic accuracy of high-frequency ultrasound in dermatology. A narrative review of the PubMed database was performed to establish the correlation between ultrasound findings and histopathologic/dermoscopic findings for cutaneous neoplasms. Articles were divided into the following four categories: melanocytic, keratinocytic/epidermal, appendageal, and soft tissue/neural neoplasms. Review of the literature revealed that ultrasound findings and histopathology findings were strongly correlated regarding the depth of a cutaneous neoplasm. Morphological characteristics were correlated primarily in soft tissue/neural neoplasms. Overall, there is a paucity of literature on the correlation between high-frequency ultrasound and histopathology of cutaneous neoplasms. Further studies are needed to investigate this correlation in various dermatologic conditions.

## Introduction

High frequency ultrasound (HFUS) has been used in clinical dermatology to visualize the physical characteristics of cutaneous neoplasms, such as depth and vascularization [1, 2]. Despite the known clinical utility of HFUS, dermoscopy followed by histopathologic diagnosis of biopsy specimens remains the gold standard for dermatologic diagnosis [1, 2]. HFUS is used as a supplementary imaging modality in conjunction with current clinical practice to noninvasively visualize deeper skin tissue at bedside, similar to how dermoscopy is used for noninvasive microscopic evaluation of lesions at bedside [1, 2]. To ensure that HFUS provides accurate visual information regarding dermatologic conditions, it is critical to elucidate the correlations between HFUS and histopathologic appearance. Thus, the purpose of this narrative review is to demonstrate the similarities between HFUS and histopathologic findings for cutaneous neoplasms.

## Materials and methods

The authors performed a search of the PubMed database with a combination of Medical Subject Headings (MeSH) and controlled terms in April 2024; no filters on language or publication date were placed. Exclusion criteria included the following: articles that did not directly make comparisons between HFUS and histopathology/dermoscopy, articles that focused on lymph node evaluation, articles discussing cutaneous metastases of primary visceral cancers, and articles that primarily featured the use of ultrasound for internal organs or did not mention ultrasound at all. No language or time filters were placed on the search query. The search query returned 495 results. 62 duplicate articles were removed. 238 articles were excluded during title and abstract screening, and 63 articles were excluded during full text screening. A total of 132 articles are included in in this narrative review. Articles were divided into four categories based on World Health Organization classification of cutaneous neoplasms: melanocytic, keratinocytic/epidermal, appendageal, and soft tissue/neural neoplasms. The aim of this review was to determine the types of cutaneous neoplasms most frequently evaluated with HFUS, as well as the diagnostic accuracy of HFUS compared to dermoscopy and histopathologic findings. The articles considered in this review are summarized in Table 1. This was not a systematic review, and this article contains no new studies with human participants or animals performed by any of the authors.

**Table 1:** Articles reviewed and key points regarding diagnostic accuracy of high-frequency ultrasound (HFUS) for various types of cutaneous neoplasms

Cutaneous neoplasm classification	Disease	Key points
Melanocytic	Melanoma	Depth on HFUS correlates with depth on histopathology
Keratinocytic/Epidermal	Basal cell carcinoma	Depth on HFUS correlates with depth on histopathology
	Squamous cell carcinoma	Depth on HFUS correlates with depth on histopathology
Appendageal	Pilomatrixoma	“Target” lesion with hypoechoic rim correlates to connective tissue capsule; Irregular echogenic center correlates to central islands of epithelial cells
Soft Tissue/Neural	Dermatofibrosarcoma protuberans	Strongest morphologic correlation between HFUS and histopathology; Oval hypoechoic dermal and hyperechoic epidermal areas with tentacle-like projections into subcutaneous fat correlates to storiform growth pattern and honeycomb invasive pattern on histopathology
	Hemangioma	Differences between RICH and NICH can be seen on both HFUS and histopathology
	Glomus Tumor	Hypoechoic masses with internal vascularity correspond to glomus cells arranged in nests and dilated vascular spaces

### Melanocytic cutaneous neoplasms

Investigation into the efficacy of HFUS for the diagnosis and treatment of melanoma demonstrates a strong correlation between histopathologic and sonographic findings. Specifically, there is a strong correlation between histopathologic and sonographic tumor thickness measurements [3–12]. In studies that specifically analyzed the correlation between measurements, the values ranged from *r* = 0.88–0.938 [3–5]. One study contradicted this strong correlation, suggesting that sonometric measurements of Breslow depth differed by 37% above and 48% below values obtained by histology in 95% of cases studied. This study limited its evaluation to malignant melanoma, excluding cases of melanoma in situ, which could account for the differences [13]. Possible causes of discrepancy between sonometric and histological values may be explained by inflammatory infiltrates, nevus cells, and adnexal structures like hair follicles [5, 14]. One study demonstrated an overall strong correlation between sonometric and histometric tumor thickness in 68 melanoma cases of *r* = 0.887 [4]. They found that thinner primary lesions, with a Breslow depth of around 1 mm, showed similar measurements 86.7% of the time [4]. Comparatively, lesions with a thickness between 1.01 and 2 mm, showed a correlation between sonometric and histologic tumor thickness only 72.2% of the time [4].

An additional study highlighted that ultrasonography tends to overestimate invasive lesions when comparing mean thickness to the complement Breslow depth from histopathology [15]. However, the data was still in strong agreement, with differences between modalities measuring 10.7% on average [15]. 

Overall, several articles provide strong statistical support regarding the correlation between HFUS and histopathology for depth of melanocytic cutaneous neoplasms. HFUS can be a useful adjunct for noninvasively determining melanoma depth [3–47]. 

Many studies highlighted the potential for ultrasound to provide auxiliary data for preoperative determination, such as determining surgical margins [48–50]. Ultimately, this could reduce the need for additional surgical interventions and improve patient outcomes. HFUS allows for immediate pre-operative measurement of tumor depth for accurate visualization of depth, which could help reduce the overall risk of recurrence [16–18]. In terms of other pigmented lesions, our search did not provide enough data to comment on the efficacy of HFUS. Several studies have investigated the diagnostic accuracy of ultrasound findings in other types of melanocytic lesions including giant congenital nevi and blue nevi, but more research is needed to confidently determine the diagnostic value of HFUS in these cases [51–55]. 

### Keratinocytic/epidermal cutaneous neoplasms

Most articles describing keratinocytic/epidermal neoplasms identified the utility of HFUS in identifying tumor margins and depth of invasion [56–61], with many detailing the diagnostic accuracy of ultrasound and its comparison to histopathology and dermoscopy.

Several articles and case reports detailed the presence of areas within suspected BCC lesions that appeared hypoechoic and oval-shaped overall [62–88]. With continued, consistent reports of these findings, physicians would be able to assess a suspected BCC lesion with HFUS, but further assessments of the reliability, sensitivity, and specificity of this sign are needed. Of all the articles that correlated histopathologic findings of BCC with HFUS, the majority found the HFUS findings accurate, but not statistically significant. A significant impact on diagnosis was only identified when evaluating benign lesions and inflammatory skin disorders; the diagnosis of malignant lesions was not significantly improved with HFUS [89, 90]. Wortsman et al. ascertained that clinical examination plus HFUS was 97% accurate in comparison to 73% accuracy with clinical examination (inculding dermoscopy and any available laboratory testing) alone [89]. From the existing data, HFUS may have some role in the evaluation of size and depth of tumors, but not diagnosis, as imaging modalities such as dermoscopy and reflectance confocal microscopy are better suited for skin cancer diagnosis [85, 90]. 

SCC was also frequently studied in conjunction with BCC. These studies found ultrasound useful to assess morphology and depth of primary tumors [68, 69, 77, 91–97]. Benign lesions, BCC, and SCC all appear hypoechoic on HFUS. However, BCC and SCC tumors have more arcuate deep borders than benign lesions [84, 98]. A case report attempted to characterize the tumor morphology, vascularization, and elasticity of SCC through ultrasound, defining it as a well-defined, hypoechogenic lesion with hypodermal hypervascularization and increased rigidity [83]. While other reviews recognized the utility of ultrasound in assessing suspicious lesions, it is not a singular diagnostic method, as histopathology, the current gold standard, is highly accessible [96, 99]. More research is needed to characterize ultrasound’s diagnostic utility in keratinocytic/epidermal neoplasms.

### Appendageal cutaneous neoplasms

Pilomatrixomas are appendageal cutaneous neoplasms defined as hair follicle tumors derived from hair matrix cells. Several studies were able to frequently identify characteristic HFUS findings that correlated accurately with histopathologic results, including one that studied over 150 pediatric patients [100–104]. On HFUS, pilomatrixomas appear as an ovoid complex mass at the dermal-hypodermal junction, presenting as a “target” lesion with a hypoechoic rim or halo around an irregular echogenic center with posterior acoustic shadowing, although this pattern was not always seen [100–102]. Specifically, in the study by Pelizzari et al., under 50% of the lesions showed the hypoechoic rim, and even fewer cases (below 30%) demonstrated a hyperechoic mass with posterior acoustic shadowing on HFUS [100]. 

Solid cystic hidradenomas, another type of appendageal cutaneous neoplasm, appear as a well-demarcated, echoluchent mass with hyperechoic protrusions on HFUS, consistent with solid material protruding from the cyst wall revealed on histopathology [105–108]. Trichilemmal tumors appear as a well-defined isoechoic nodule with hypoechoic curved lines that correspond to stroma between lobules on histopathology [109, 110]. 

#### Soft tissue/neural cutaneous neoplasms

Dermatofibrosarcoma protuberans (DFSP) is a rare soft tissue tumor that classically presents as a firm plaque on the trunk due to overgrowth of the dermis or subcutaneous fat. DFSP perhaps had the most strongly correlated HFUS findings with its histopathology [111–113]. One case report noted irregular infiltration of the tumor into adipose tissue along with significant hypervascularity [114]. More recently, several studies have described DFSP as oval hypoechoic dermal and follicular induction indicated by hyperechoic epidermal areas with tentacle-like projections into the subcutaneous fat [115, 116]. Hypoechoic areas with a storiform growth pattern on histology correlated with hyperechoic areas with a histologic honeycomb invasive pattern [114, 115, 117, 118]. Of note, one case study reported a DFSP originally mistaken on ultrasound for a lipoma; [119] thus, high clinical suspicion should still be used in cases where DFSP is on the differential.

In pediatric dermatology, HFUS was identified as a remarkable imaging modality to assess infantile hemangiomas, the most common vascular tumors of infancy [120]. The HFUS images not only provided valuable insights into the anatomical characteristics of hemangiomas, but also paved the way for histopathological correlations. Histopathological examination of infantile hemangiomas revealed dilated blood vessels, endothelial cell proliferation, and varying degrees of fibrous stroma within the lesions, aligning with the ultrasound characteristics of well-defined hypoechoic masses with peripheral hyperechoic halos and internal vascularity [120]. 

Doppler ultrasound is a valuable modality used to visualize blood flow, providing crucial insights into the characteristics of both rapidly involuting congenital hemangiomas (RICH) and non-involuting congenital hemangiomas (NICH). These vascular tumors are typically confined to subcutaneous fat and exhibit a heterogeneous appearance with occasional calcifications, along with multiple arteries and veins displaying high-velocity blood flow [121–127]. Histopathological analysis of RICH reveals well-defined, variably sized lobules containing small capillaries and prominent draining vessels in the dermis and subcutaneous tissue, while NICH presents well-defined large lobules of capillaries and draining channels that appear more dilated, larger, and thicker-walled. Additionally, interlobular areas in NICH contain fibrous tissue with prominent arteries, dilated and dysplastic veins, and arteriolobular and arteriovenous fistulae, as summarized in Table 2 [53, 54]. The histopathologic findings align closely with the observations from Doppler ultrasound, with NICH often exhibiting more vascular shunts than RICH [121, 128]. 

**Table 2:** Comparison between characteristics of rapidly involuting congenital hemangiomas (RICH) and noninvoluting congenital hemangiomas (NICH) as seen on both high-frequency ultrasound and histopathology

Vascular neoplasm type	Lobule size	Capillary size	Additional characteristics
RICH	Variable	Small	Draining channels appear more dilated, larger, and thicker-walled
NICH	Large	Large	Interlobular areas contain fibrous tissue with prominent arteries, dilated and dysplastic veins, and arteriolobular and arteriovenous fistulae

Moving beyond congenital hemangiomas, ultrasound has also proven valuable in evaluating other types of vascular tumors. For instance, a relatively uncommon type of hemangioma known as spindle cell hemangioma was evaluated using ultrasound in a case report by Kamiya et al. [129] The histopathologic findings revealed multifocal lesions consisting of dilated and congested vascular spaces and fascicles of spindle-shaped cells. Ultrasound examination showed a multinodular lesion with blood-flow signals in the dermis without contiguous veins, which corresponded with the rich vasculature observed on histopathology. [129]

A few articles were found in the literature review that specifically discussed the correlation between histopathology and HFUS for glomus tumors. The hypoechoic masses with internal vascularity, as visualized on ultrasound, were consistent with the presence of glomus cells arranged in nests and dilated vascular spaces observed in histopathological analysis [130–132]. These compelling findings not only emphasize the diagnostic value of ultrasound imaging in detecting and differentiating glomus tumors but also underscore the synergistic relationship between HFUS and histopathology.

## Discussion

The current quality of evidence regarding the diagnostic accuracy of HFUS imaging in the diagnosis and management of dermatological conditions is low. By comparing ultrasound characteristics with histopathological results, dermatologists can improve diagnostic accuracy and gain a deeper understanding of how the HFUS appearance correlates with underlying histological features of skin neoplasms. Review of the literature revealed there are stuudies on the use of HFUS in dermatology, but a relative paucity of literature that rigorously assesses its use to test prospective and diagnostic or prognostic accurancy. Furthermore, there is an overall lack of evidence in the literature regarding the ability of HFUS to differentiate between in-situ melanomas and nevi. Benign nevi may appear the same as in-situ melanomas on HFUS due to lack of significant differences in depth; future studies may be warranted to investigate this.

It is important to note the possibility that small-diameter melanomas can be invasive and large-diameter melanomas can be in-situ, and HFUS can non-invasively differentiate between these because of its strong correlation to Breslow depth. Similarly, tumor depth of non-melanoma skin cancers may not necessarily be directly correlated with clinical diameter, further supporting the use of HFUS to determine concerning morphologic features and their effect on surrounding structures. A small-diameter non-melanoma skin cancer may have invasive characteristics that can be seen on HFUS.

There is evidence in the literature supporting the use of reflectance confocal microscopy for non-invasive diagnosis because of its strong correlation to histopathology [36, 37]. However, it is important to note that unlike HFUS, reflectance confocal microscopy lacks depth resolution. Thus, a combination of HFUS and reflectance confocal microscopy may be helpful for non-invasive diagnosis of cutaneous neoplasms. Future studies may help to elucidate the efficacy and practicality of using these imaging methods in conjunction.

Cutaneous neoplasms with the most frequently reported data on the diagnostic accuracy of HFUS include melanoma, BCC, SCC, and DFSP. Regarding melanoma and non-melanoma skin neoplasms, there is more documentation on the strong correlation between HFUS and histopathology regarding the depth of a cutaneous neoplasm. There are fewer articles that discuss the HFUS-histopathology correlation regarding the morphological characteristics of the neoplasm itself. Key takeaway points from this review are summarized in Table 1.

A key limitation of this narrative review is the lack of literature showing the use of HFUS to diagnose rare types of melanoma, including desmoplastic, nested, dermal, and mucosal. There is a wide variation in the amount of pigmentation in both melanomas and non-melanoma skin cancers. Thus, HFUS may be useful for elucidating concerning features of cutaneous neoplasms regardless of the amount of pigmentation. Based on the available evidence of HFUS use for common types of melanoma, HFUS also has the potential to assist with diagnosis of rare melanoma types. Further studies of HFUS in rare melanoma types may be warranted.

In conclusion, the current literature reveals strong evidence about about the ability of HFUS to accurately determine the size of cutaneous neoplasms, which could be helpful for procedural planning, but weaker evidence for morphological characteristics of cutaneous neoplasms. In some cases when histopathology is not regularly employed, such as hemangiomas, HFUS can provide visual information to guide diagnosis and management. Additional studies are warranted to rigorously investigate the correlation between histopathology and HFUS findings for skin conditions.

## Data Availability

No datasets were generated or analysed during the current study.
